# Prevalence of Cesarean Section and Its Indications in a Tertiary Care Hospital

**DOI:** 10.31729/jnma.4282

**Published:** 2019-04-30

**Authors:** Smrity Maskey, Manisha Bajracharya, Sunita Bhandari

**Affiliations:** 1 Department of Obstetrics and Gynecology, KIST Medical College and Teaching Hospital, Imadole, Lalitpur, Nepal

**Keywords:** *cesarean section*, *fetal distress*, *prevalence*

## Abstract

**Introduction:**

Cesarean section is a surgical procedure performed to deliver fetus through abdominal route. Increasing rate of cesarean section worldwide is an alarming concern for public health and obstetricians due to increase in financial burden and risk to health of the mother in comparison to vaginal delivery. The aim of the study was to find the prevalence of cesarean section and its most common indication in a tertiary care hospital.

**Methods:**

This descriptive cross-sectional study was done in a tertiary care hospital, from July 2016 to June 2018 after taking ethical clearance from institutional review board registration number 38970/062/063. Convenience sampling was done. Data was collected and entry was done in microsoft excel, point estimate at 95% Confidence Interval was calculated along with frequency and proportion for binary data.

**Results:**

Out of total deliveries conducted, 862 (36.8%) were Cesarean section deliveries, 1477 (63.1%) were vaginal deliveries, and 12 (0.51%) were instrumental deliveries. Prevalence of Cesarean section is 862 (36.8%) at 95% Confidence interval (35–39). Mean age±SD of delivering mother was found to be 26.1+0.25 years. Primi cesarean section was more than repeat cesarean section. Most common indication of cesarean section was fetal distress 243 (28%) followed by previous cesarean section 165 (18%), non-progress of labour 106 (12%), oligohydramnios 59 (7%), malpresentation 59 (7%), cephalo pelvic disorders 52 (6.5%) and hypertensive disorder in pregnancy 33 (4%).

**Conclusions:**

Prevalence of cesarean section in a tertiary care hospital is high compared to WHO data. The most common indication of cesarean section are fetal distress and previous cesarean section.

## INTRODUCTION

Cesarean section (CS) is a surgical procedure performed to deliver fetus through abdominal route. CS is one of the oldest operation in surgery.^1^ The objective of CS in ancient world was for post mortem delivery but in modern medicine it has saved many mothers and babies.

Increasing rate of cesarean section worldwide is an alarming concern for public health and obstetrician due to increase in financial burden and risk to health of the mother in comparison to vaginal delivery.^2^ This increase rate in developed country is due to early diagnosis of fetal distress by continuous use of electronic fetal monitoring and malpresentation, however the cause of increase rate of CS in developing countries like Nepal is not clear.^3,4^

The aim of the study was to find the prevalence of cesarean section and its most common indication in KIST Medical College and Teaching Hospital.

## METHODS

It is a descriptive cross-sectional study done in KIST Medical College and Teaching Hospital, Imadole, Lalitpur, from July 2016 to June 2018. Ethical clearance was taken from institutional review board of KIST Medical College and Teaching Hospital with registration number 38970/062/063. Study population were pregnant women who were admitted and delivered in the hospital. Inclusion criteria for participants in the study is patients who had their deliveries at the study site and exclusion criteria for participants in the study is patients who did not give consent for the study, patients who were admitted to the hospital for observation and cases admitted for gynecological treatment.

Data collection was done by filling self-structured performa designed for the study. Data was collected throughout the study period to meet the sample size for the study.

Convenience sampling was done and sample size was calculated using the formula,


$$\begin{array}{l} \text{n}={\text{Z}}^{2}\times (\text{p}\times {\text{q)/d}}^{2}\\ =1.96^{2}\times (0.4\times 0.6)/0.02^{2}\\ =2304.96\\ =2305 \end{array}$$

where,

n = sample size
p = prevalence of CS (educated guess i.e. 40%).
q = 1-p
d = margin of error (2%).
Z = 1.96 at 95% CI.

After taking non-respondent rate of 5%, total sample size is calculated to be 2421.

Selection and information bias has been minimized as possible. Data entry was done in microsoft excel, point estimate at 95% CI was calculated along with frequency and proportion for binary data and analysis was done.

## RESULTS

**Figure 1.**
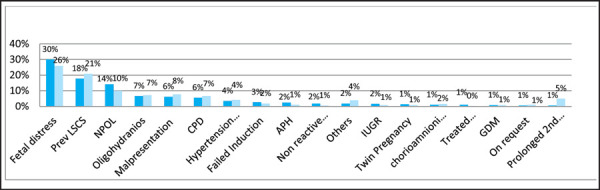
Indications of total cesarean section (emergency and elective).

There were 2339 deliveries done in the hospital. Out of total deliveries conducted, 862 (36.8%) were CS deliveries, 1477 (63.1%) were vaginal deliveries and 12 (0.51%) were instrumental deliveries. Prevalence of CS is 862 (36.8%) at 95% of CI (35–39). Among cesarean deliveries, 128 (27%) and 251 (34%) were elective CS; 355 (73%) and 128 (66%) were emergency CS in two years respectively. The most common indication of CS in the two year study was fetal distress 243 (28%) followed by previous cesarean section 165 (18%), non-progress of labor 106 (12%), oligohydramnios 59 (7%), malpresentation 59 (7%), cephalo pelvic disorders 52 (6.5%) and hypertensive disorder in pregnancy 33 (4%) (Figure 1).

**Figure 2.**
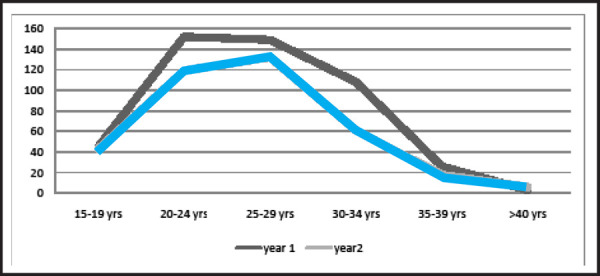
Age distribution of cesarean section.

Most mothers who had cesarean section were of 25–29 years age group and the mean age ±SD was 26.1±0.25 years (Figure 2).

**Figure 3.**
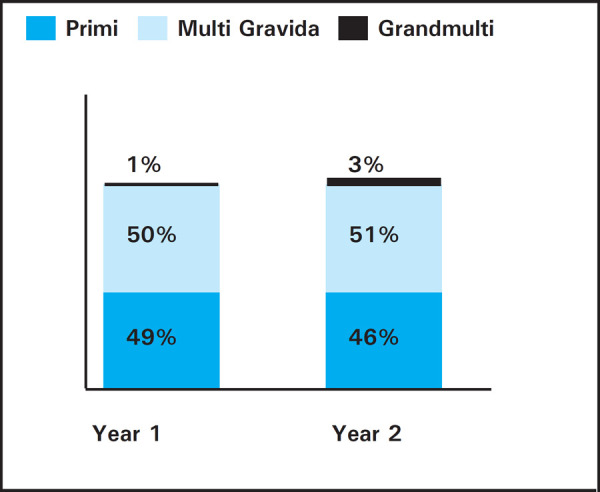
Showing parity in two years.

Most females who came to our facility were 241 (50%) and 194 (51%) multi-gravida followed by 235 (49%) and 175 (46%) primi-gravida, along with 7 (1%) and 10 (3%) grandmulti-para in year 1 and 2 respectively (Figure 3).

**Figure 4.**
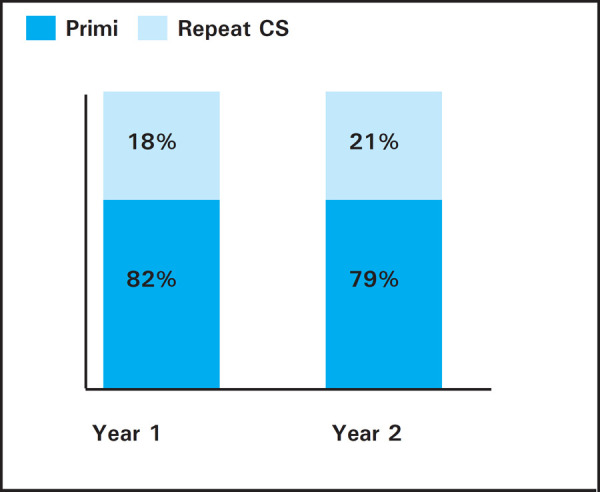
Showing of Primi and Multiple CS in two years.

The study revealed that 397 (82%) and 300 (79%) primi-cesarean deliveries were markedly more than 86 (18%) and 79 (21%) repeat cesarean section in respective year 1 and 2 (Figure 4).

## DISCUSSION

WHO report in 1985 suggested that optimal population range for cesarean section rates is between 5% and 15%.^5,6^ This reference range is very low as compared to our study where CS rate is 36.8%. On the other hand, Nigeria, a developing country like ours have a CS rate of only 11.8%.^7^ This could be due to recent increase in hospital delivery rate in Nepal, 59% as stated in NDHS 073/074.^8^ Study by S. Subedi also supports our finding.^9^ In studies conducted in other centres of Nepal like Kathmandu Medical College, Patan hospital, Kirtipur hospital showed CS rate of 45.8%,^10^ 41.9%,^11^ and 50.9%^12^ respectively which is more than our study. Hence the CS rate is rising in Nepal as well ^9,13–15^ from 18.8% in mid-western region to 37.7% in eastern Nepal. The rate of CS in respective years in our study is 37.8% and 35.7% which is almost same. However, study done by Mellisa et al. in South East Asian country shows increasing trend of CS which is in contrast to our study.^16^ This discrepancy could be due to small sample size and short study duration in our study.

Mothers giving birth were from 25–29 years age group with the mean age of 26.1±0.25 years in our study which supports the study done in Nigeria where mean age was 27.4 years.^7^ The 25–29 years age group was maximum child bearing age group in our study, and also in other studies done in Nepal,^15^ India^17^ and Bangladesh.^18^ Increasing maternal age was associated with increased odd of CS in urban India.^16^ Study done by K. Dhakal in mid-western Nepal shows age distribution more in 2024 years,^14^ which is different from our study. This may be due to geographical and cultural variation for age of marriage in Nepal.

Most patients who underwent CS were multi-gravida in our study which is consistent with the studies done in Nigeria,^7^ and in contrast to study done by S. Subedi.^9^

While comparing the primi and repeat CS, our study showed exceeding high primi-CS rate which is very alarming for future repeat CS. This is also true for other studies done in Nepal. ^9,14^ This can be because there is no practice of trial of vaginal birth after CS (VBAC) and breech presentations irrespective of parity undergo CS. CS are also done on patient's request which is increasing nowadays.

Emergency CS was more than Elective CS in both years in our study as KIST is a tertiary care referral hospital where complicated cases are referred for CS delivery.

Most common indication of CS in most part of the world and Nepal are fetal distress followed by previous CS and malpresentation. In our study and tribal study of India, indication of CS for fetal distress is very high.

Our study showed 30% in year 1, 26% in year 2 data of foetal distress similarly 31.20% in tribal and 30.60% in non-tribal study conducted in India.^17^ Indication of CS for foetal distress showed data as 8.1% in year 1 and 10% in year 2 in BPKIHS,^13^ 9.6% in Nigeria^7^ and 21% in Bangladesh.^18^ This huge disparity is because in other studies fetal distress and meconium stained liquor are kept in different category where as in our studies they are kept in same headings.

Cesarean section rate and its trend is increasing worldwide so does in Nepal. Of the many contributing factor for increase this CS rate, the important two causes which cannot be ignored but has no data to support are firstly the legal complication against a doctor now-a-days that everyone fears and no obstetrician wants to take risk involved with vaginal delivery. Secondly, with current small family trend people want just one or two babies and that also via CS due to fear of labor pain. In view of above causes the trend in rise of CS need more prospective prolonged study along with maternal and perinatal outcome.

## CONCLUSIONS

Prevalence of cesarean section in KIST Medical College and Teaching Hospital is high compared to WHO data. The most common indication of cesarean section are fetal distress and previous cesarean section.

## Conflict of Interest

**None.**
